# Liver Echinococcosis complicated with Lesions of Bile Ducts in Azerbaijan

**DOI:** 10.5005/jp-journals-10018-1183

**Published:** 2016-12-01

**Authors:** Rauf M Aghayev

**Affiliations:** Department of Surgical Diseases-II, Azerbaijan Medical University, Baku, Azerbaijan

**Keywords:** Bile ducts, Bile duct lesion, Complications, Cystobiliary fistulas, Laser irradiation, Liver echinococcosis, Liver hydatid disease, Surgery.

## Abstract

A total of 302 patients with liver echinococcosis (LE) complicated by lesions of bile duct during 1988 to 2015 were analyzed. The patients were divided into two groups. In the first group, 227 patients were included with lesions of segmental bile ducts in the form of cystobiliary and bilio-bronchial fistulas. In the second group, 75 patients with lesions of hepatic bile were enrolled. Diagnosis of LE and its complications was made by ultrasonography and computed tomography (CT). Surgical treatment included echinococcectomy with complete liquidation of a residual cavity (35.1%), echinococcectomy with suturing (46.0%), external drainage of a residual cavity (7.3%), pericystectomy (8.6%), and resection of a liver (3.0%). Postoperative complications related to operative intervention developed in 29 patients. Lethal outcomes took place in 6 cases (2.0%). The analysis has shown that the form of bile duct lesions, methods of surgical operation for liquidation of hydatid cysts, and its biliary complications had influenced the treatment outcome. More optimal results are received after complete liquidation of residual cavity in different ways and suturing of cystobiliary fistulas during operation. For prevention of complications related to the presence of residual cavity, laser processing of walls of a residual cavity by ozonized 17% hypertonic solution of NaCl was used and this procedure showed obvious (p < 0.05) advantages.

**How to cite this article:**

Aghayev RM. Liver Echinococcosis complicated with Lesions of Bile Ducts in Azerbaijan. Euroasian J Hepato-Gastroenterol 2016;6(2):125-130.

## INTRODUCTION

Liver echinococcosis (LE) is a serious parasitic disease, which remains a serious medical and economic problem in many countries around the world, including Azerbaijan.^1-3^ Azerbaijan is included among the countries with high incidence of LE (about 1 patient per 5000–10000 inhabitants).^4^ Despite considerable achievements in liver surgery, unfortunately, the number of patients affected does not tend to decrease. As a result of late diagnosis, surgical treatment is needed in considerable numbers of patients and postoperative lethality may reach as high as 5 to 10%.^1,5,6^

One of the most dangerous complications of the LE is a rupture of cysts in bile ducts. However, there is paucity of information on bile ducts lesions in patients with LE and it may vary from 1.3 to 55%.^7-9^ According to some researchers, sooner or later, 90% of all liver hydatid cysts are complicated by bile duct lesions.^10^

Although surgery remains the gold standard treatment for hydatid liver disease, the frequency of recurrence of LE may be 3 to 38%^11-13^ and the frequency of postoperative complications may also vary from 22.1 to 53%.^14-16^ The study presented here provides an update of therapy of LE complicated with bile duct lesions in Azerbaijan.

## MATERIALS AND METHODS

The presented work is based on the retrospective analysis of surgical treatment of 302 patients with LE, complicated by lesions of bile ducts, which were operated at Scientific Center of Surgery, Azerbaijan Medical University during the period from 1988 to 2015. During this period, 1,314 patients with LE were treated. A total of 302 patients of LE with bile duct lesions were enrolled for this study. The age of patients varied from 8 to 86 years with an average age of 39.7 years.

The patients were divided into two groups (Table 1). In the first group, 227 patients were included and the second group contained 75 patients. The details of the lesions have been shown in Table 1. Most of the patients had the disease duration more than a year, and 23.2% of them had been suffering from 5 years or more.

**Table 1:** **Table 1:** Character and frequency of main lesions of bile ducts in patients with liver echinococcosis

				*Number of patients*			
*No.*		*Character of lesions*		*Total*		*%*	
I		Segmental bile ducts		227		75.2	
		(a) Cystobiliary fistulas		220		72.9	
		(b) Bilio-bronchial fistulas		7		2.3	
II		Hepatic bile ducts		75		24.8	
		(a) Rupture into bile ducts		35		11.6	
		(b) Compression of bile ducts		34		11.3	
		(c) Cicatricial strictures of bile ducts		6		1.9	
		Total		302*		100	

*In 23 (7.6%) patients simultaneous lesions of bile ducts noted

In most of the patients (230–76.2%), the hydatid cysts were localized in different segments of the right lobe. Both lobes of a liver were damaged in 51 (16.9%) patients. Hydatid cysts were complicated with partial or full calcification of fibrous capsule in 13.9% and with suppuration of cyst contents in 27.4%.

The diagnosis was based on clinical, laboratory, and investigational approaches. Ultrasonography was the screening method of choice. Computed tomography (CT) scan was performed at 86 patients in the preoperative period and has the highest sensitivity of imaging of the cysts (100%). Magnetic resonance imaging (MRI) scan was done in 37 patients.

One of highly informative diagnostic tools was using direct contrast methods. Endoscopic retrograde cholangiopancreatography (ERCP) as a method of investigation was performed in 12 patients and has allowed revealing pathological changes in bile ducts in most patients. Intraoperative cholangiography through a cystic drain or a T-tube was performed in suspected intrabiliary rupture and bile duct obstruction in 43 patients.

The complex of prophylaxis of postoperative complications begun from the moment of preparation of patients to the radical operation (i/v injection of 200 mL of the 0.9% NaCl solution with ozone of 4–6 mg/L), and also antioxidant therapy (injection of 30% solution of tocopherol-acetate in a dose of 20 mg/kg).

For this purpose, for prevention of dissemination of serous surfaces of the patient with germinal elements of a parasite, we applied original methods of antiparasitic isolation during puncture of hydatid cysts and removing of their content.

**Fig. 1:**
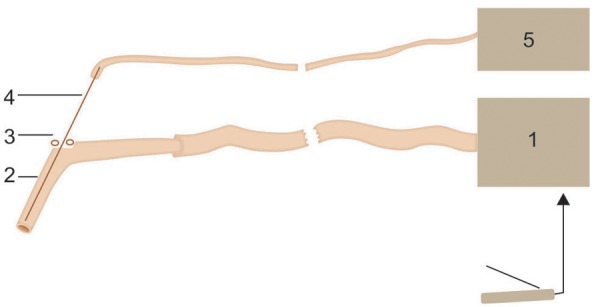
Principle of operation of the device for removal of cyst’s contents accepting capacity; 1 – the accepting capacity for remote contents of a cyst; 2 – bent tube; 3 – lateral aperture; 4 – needle for puncture; 5 – source of vacuum

**Figs 2A and B:**
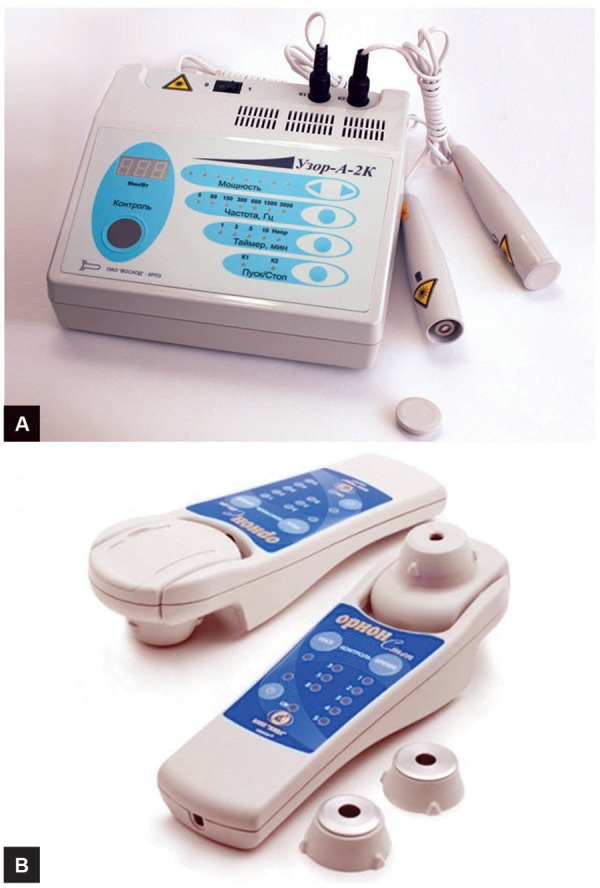
Laser apparatus for processing of residual cavities (A) “Uzor” and (B) “Orion”

Special devices were used for puncture and drainage of echinococcal cysts (Fig. 1). For prevention of complications connected with the presence of residual cavity, laser therapeutic devices “Uzor” and “Orion” were used, as shown in Figures 2A and B. In addition, cavities of 51 patients with ozonized 17% hypertonic solution of NaCl (with concentration of ozone 40–60 mg/L and with an exposition of 4–8 minutes). The details of the executed surgical operations and numbers of patients subjected to each type of operations has been presented in Table 2.

**Table 2:** **Table 2:** Type of operations

*Type of operations*		*Total*		*%*	
Echinococcectomy with complete liquidation of residual cavity		106		35.1	
(a) Without excision of fibrous capsule		41		13.6	
(b) With partial excision of fibrous capsule		65		21.5	
Echinococcectomy with suture appliance and external drainage of residual cavity		139		46.0	
(a) Without excision of fibrous capsule		48		15.9	
(b) With partial excision of fibrous capsule		91		30.1	
Echinococcectomy with external drainage of residual cavity		22		7.3	
Pericystectomy		26		8.6	
Resection of liver		9		3.0	
Total		302		100	

For estimation of the efficiency of the offered method in processing of walls of a residual cavity, we carried out comparative bacteriological and morphological intra- and postoperatively researches in two groups of patients.

Smears for bacteriological investigations during operation were taken twice: Before and after corresponding processing. Before the processing of a residual cavity, pathological microflora was found almost in all patients of both groups.

## RESULTS

All 302 patients underwent surgery. The surgical method was determined from the number and location of cysts, their connection with bile ducts, and the presence of preoperative complications. The diagnosis of echinococcosis and its biliary complications continued also intraoperatively. Visual examination allowed to detect cystobiliary fistulas in 168 patients.

The details of surgical procedures and the numbers of patients subjected to these procedures have been shown in Table 2. In some patients, we failed to conduct adequate visual revision of the residual cavity, located in a remote region of the liver, although there were clear evidence of the presence of bile in it. For determination of small size bile fistulas on a fibrous capsule, we intraoperatively applied express method (5–10 seconds) that detects cystobiliary fistulas by means of specific chromatic reaction. Sites with observed chromatic reaction (bile availability) were sewed with atraumatic needles.

**Graph 1:**
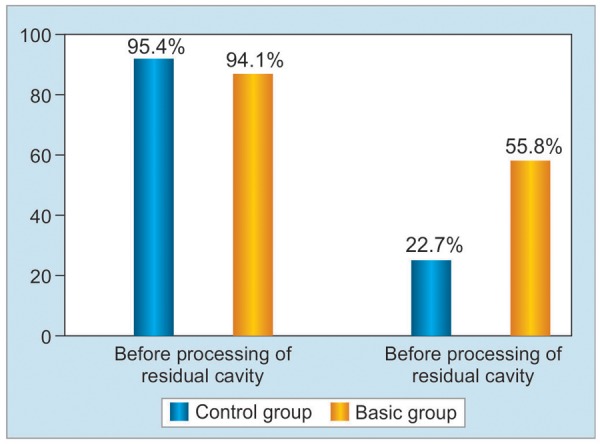
Results of bacteriological investigations before and after processing of residual cavity

Smears taken from walls of a fibrous capsule were checked for *Staphylococcus epidermidis, Staphylococcus aureus, Escherichia coli, Klebsiella,* or associations of the listed bacteria. After processing of a residual cavity by laser and ozonized solution, microbe contamination was detected in 22.7% of patients, while in patients of the control group – in 55.8% cases, i.e. 2.5 times more frequent, than in the basic group, p < 0.01 (Graph 1).

**Figs 3A to D:**
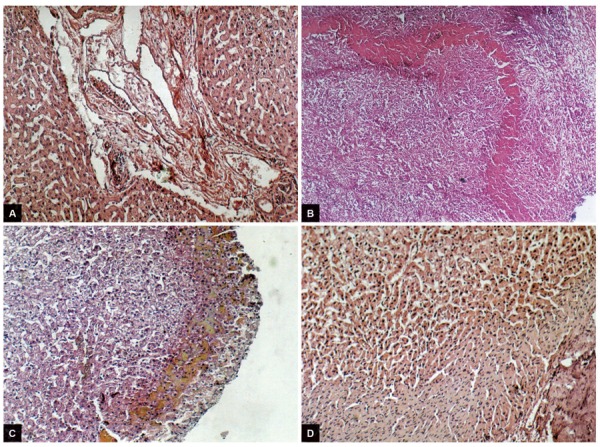
(A) Microscopy of liver tissue after processing of cavity with laser and ozonized solutions. Intra- and interlobular stromal infiltration by neutrophils, dystrophy of hepatocytes; (B) expanded and deformed vessels in fibrous capsule and in pericapsular fibrous layer, lymphocytic infiltration of hepatocytes; (C) microscopic view of microgranulomas and cellular infiltration in a fibrous capsule of cyst against vascular reaction after laser irradiation of cyst cavity; and (D) hystoarchitectonic of liver parenchyma. “Opacification” of parenchyma, deformation of hemocirculation, lymphoid infiltration in interlobular layers. H&E stain (×150)

Histological analysis has revealed fibrous capsule and pericapsular fibrous growths inclining into parenchyma of liver. In patients of this group, epithelioid and huge multinuclear phagocytes are registered much more often. The microcenter of necrosis was defined only in one case. This circumstance allows to assert an absence of expressed necrotic effect of laser treatment on liver parenchyma (Figs 3A to D).

**Figs 4A and B:**
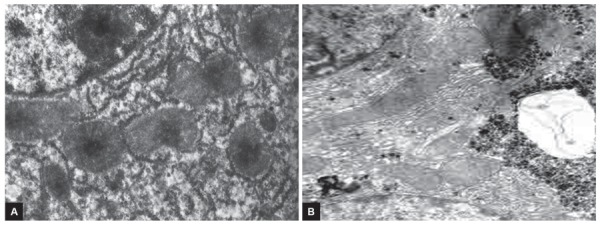
Histochemical investigation after processing of residual cavity with laser and ozonized solutions: (A) Hyperplasy and hypertrophy of endoplasma in perinuclear zone of centolobullar hepatocyte; and (B) ultrastructural view of mitochondrions and organoids in perinuclear zone of hepatocyte. Partial focal violations of mitochondrions integrity (×3000)

Figures 4A and B shows the histochemical analysis, after processing of residual cavity with laser and ozonized solutions. The nature of postoperative complications has been shown in Table 3. Postoperative complications developed in 53 (17.5%) patients, and in 29 (9.6%) of them were connected to a character of operative intervention (specific complications). Relaparotomy were performed in 7 patients (2.3%). Lethal outcomes took place in 6 cases (2.0%), caused by acute hepatorenal failure on the background of purulent cholangitis (2 patients), sepsis after subdiaphragmal abscess (1), cardiovascular failure (2), and pneumonia (1).

**Table 3:** **Table 3:** Nature of postoperative complications

*No.*		*Complications*		*Number* * of cases %*		*Number* * of reoperations %*		*Lethal* * outcomes %*	
I		Specific complications		29 (9.6)		7 (2.3)		3 (1.0)	
		Suppuration of sutured residual cavity		3 (1.0)		1 (0.3)			
		Suppuration of drained residual cavity with formation of external biliary and purulent fistula		17 (5.7)		–			
		Hemorrhage in residual cavity		1 (0.3)		1 (0.3)			
		Biliary peritonitis		1 (0.3)		1 (0.3)			
		Cholangitis, acute hepatorenal failure		4 (1.3)		1 (0.3)		2 (0.7)	
		Subdiaphragmal and subhepatic abscesses		3 (1.0)		3 (1.0)		1 (0.3)	
II		Nonspecific complications (pneumonia, cardiovascular failure, etc.)		24 (7.9)		–		3 (1.0)	
		Total		53 (17.5)		7 (2.3)		6 (2.0)	

## DISCUSSION

There is a lot of disputable questions in the decision of a problem of successful surgical treatment of LE, and the main purpose of the decision is to improve the methods of operative treatment, allowing to decrease number of complications and to reduce a number of relapses of the disease.

Surgery is the only method of treatment of LE; however, despite significant achievements in surgery of the liver, improvement in methods of instrumental diagnostics, introduction of new technical means, effective surgical treatment of LE is yet to be optimized. Besides, some patients do not respond to surgery.^7,10,17^

The analysis on the reasons of development of adverse results has shown that the results of treatment were influenced by the form of bile duct lesions, methods of surgical operation for liquidation of hydatid cysts, and its biliary complications. More optimal results were received after complete liquidation of residual cavity in the different ways and suturing of cystobiliary fistulas during operation.

Analysis of postoperative period has shown that number of hospital days depended on a character of applied operation. Quantity of stay in hospital was less (10.2 ± 3.4 days) in group of patients who underwent echinococcectomy with complete liquidation of residual cavity (including pericystectomy and liver resection). At the same time, the quantity of hospital days was 20.4 ± 2.1 days (p < 0.001) among the patients at whom operation was finished with external drainage of a residual cavity.

One of the most important problems of surgical treatment of patients with LE is high risk of development of purulent-biliary complications.^14,18,19^ For prevention of development of infectious and biliary complications after echinococcectomy and acceleration of the processes of reparation in residual cavity, we also used laser and ozone therapy in postoperative period.

Laser was applied within a week after operation. Laser apparatus was used in two operating modes: Subcutaneous (influence on a zone of projection of a removed cyst) and through the drainage left in a cysts cavity with the help of specially made device. At the same time, in all patients with external drainage of a residual cavity for prevention of development of purulent complications, we daily washed out cavity by means of the ozonized (concentration of ozone 40–60 mg/L) NaCl solution.

Objective data (30 patients with a traditional technique of treatment and 30 with the use of complex laser radiation and ozonotherapy during and after operation) have shown obvious (p < 0.05) advantages of the last, regarding the intensity of reduction of a residual cavity and the progressive decrease (approximately twice) of a discharge from a cyst cavity. Besides, total number of purulent-biliary complications of a residual cavity at patients with radiation of cyst cavity carried out intra- and postoperatively was observed only in 2 (6.6%) cases, whereas in the control group of patients these complications took place in 5 (16.7%) observations, i.e., 2.5 times more often (p < 0.01).

For studying the advantages of the offered methods in prevention of complications in patients with external drainage of residual cavity, we performed postoperative bacteriological investigations of exudates. There have been variability in degree of bacterial contamination in different groups (control group – 18 patients with traditional technique of treatment and main group – 18 patients with the use of laser irradiation and ozonotherapy during and after operation) also were noted in postoperative period. Microflora was found in patients of the control group nearly 5 times more often than in patients of the main group (5.5 and 26.4% respectively).

Based on our experiences in vast numbers of patients with LE and bile duct involvement, it seems that proper diagnosis and time intervention with modern technologies are mandatory.
